# Cut from the same cloth? investigating the personality of interventional and surgical cardiovascular specialists

**DOI:** 10.1007/s00423-025-03874-7

**Published:** 2025-10-23

**Authors:** Vincent Q. Sier, Merel J. Verhagen, Maurits Zegel, Roderick F. Schmitz, Carla S.P. van Rijswijk, Jan van Schaik, Eduard J.E.T. Schmidt, Jaap F. Hamming, Abbey Schepers, Inez J. Wijdh-den Hamer, Mark C. Burgmans, Jesper Hjortnaes, Joost R. van der Vorst

**Affiliations:** 1 Department of Surgery, Leiden University Medical Center, Leiden, 2300 RC the Netherlands; 2 Department of Surgery, Haga Hospital, The Hague, 2545 AA the Netherlands; 3 Department of Cardiothoracic Surgery, University Medical Center, Groningen, Groningen, 9713 GZ the Netherlands; 4 Department of Surgery, Groene Hart Hospital, Gouda, 2800 BB the Netherlands; 5 Institute of Public Administration, Leiden University, Hague, 2501 EE the Netherlands; 6 Department of Radiology, Leiden University Medical Center, Leiden, 2300 RC the Netherlands; 7 Department of Cardiothoracic Surgery, Leiden University Medical Center, Leiden, 2300 RC the Netherlands

**Keywords:** Surgeon personality, Big five personality, Team dynamics, Aortic team, Multidisciplinary team, Aortic surgery

## Abstract

**Purpose:**

Considering that the treatment of patients with complex vascular disease requires multidisciplinary collaboration in teams and personality traits can impact team dynamics, we investigated the personality structures of vascular surgeons, cardiothoracic surgeons, and interventional radiologists using the validated big five model.

**Methods:**

A cross-sectional study utilizing the validated Big Five Inventory-2 (BFI-2) questionnaire. Corrected one-way analyses of variance were performed to compare personality domain scores between the specialist groups. Questionnaires were distributed among all Dutch general surgery departments and through the Dutch Societies for Interventional Radiology (NVIR) and Cardiothoracic Surgery (NVT). A total of 224 specialists participated: 78 interventional radiologists (mean age 48.2y, 20.5% female), 41 cardiothoracic surgeons (mean age 49.2y, 26.8% female), and 105 vascular surgeons (mean age 49.9y, 17.1% female).

**Results:**

For the personality domains agreeableness and negative emotionality, differences were observed between the three specialist groups, while the scores for open-mindedness, conscientiousness, and extraversion were similar. In particular, cardiothoracic surgeons scored higher on agreeableness (4.13 vs. 4.00; *p* = .046) relative to interventional radiologists. Vascular surgeons had lower scores on negative emotionality relative to interventional radiologists (2.00 vs. 2.20, *p* = .007). Differences at the facet-level were present in four of the five personality domains between interventional and surgical specialists, including sub-traits such as compassion, creative imagination, and assertiveness.

**Conclusion:**

While vascular surgeons, cardiothoracic surgeons, and interventional radiologists are involved in shared cardiovascular care pathways, they display nuanced differences across domains and more granular facets of personality. These findings lay the foundation for studies on self-awareness and interprofessional collaboration in shared clinical pathways through joint understanding of personality in multidisciplinary teams.

**Supplementary Information:**

The online version contains supplementary material available at 10.1007/s00423-025-03874-7.

## Introduction

Personality traits, referring to enduring patterns of thoughts, feelings, and behaviors, play a crucial role in professional development and personal interactions. Average personality traits of various professional groups within and beyond medicine have been documented, often using the validated five-factor taxonomy, a structure of traits captured in five domains. A well-known instrumental version of this “big five” model identifies the five domains as: open-mindedness, conscientiousness, extraversion, agreeableness, and neuroticism/negative emotionality. Importantly, these five domains are further built up by “facets”, sub-categories that together may give more granular insight into personality (Table 1).

**Table 1:** Overview of big five domains and facets

Domain	Description [35]	Facets[13, 14]
Open-mindedness	Describes the breadth, depth, originality and complexity of an individual’s mental and experiential life	Intellectual curiosity
		Aesthetic sensitivity
		Creative imagination
Conscientiousness	Describes socially prescribed impulse control that facilitates task- and goal-directed behavior, such as thinking before acting, delaying gratification, following norms and rules, and planning, organizing and prioritizing tasks	Responsibility
		Organization
		Productiveness
Extraversion	Implies an energetic approach toward the social and material world and includes traits such as sociability, activity, assertiveness and positive emotionality	Energy level
		Sociability
		Assertiveness
Agreeableness	Contrasts a prosocial and communal orientation toward others with antagonism and includes traits such as altruism, tender-mindedness, trust and modesty	Trust
		Compassion
		Respectfulness
Negative emotionality	Contrasts emotional stability and even-temperedness such as feeling anxious, nervous, sad and tense	Emotional volatility
		Anxiety
		Depression

Current research on the personality of medical specialists has often focused on specific subspecialties, on contrasts with the general population or has drawn general pooled comparisons between surgical and non-surgical specialty groups. For instance, it has been shown that surgeons generally have high levels of conscientiousness and emotional stability [1, 2]. Although these and other studies have shown general similarities and differences between surgical, medical, or supporting specialties [3, 4], the potential value of personality insights in specialty groups who directly participate in shared multidisciplinary clinical teams has not yet been investigated.

From a personality perspective, character traits affect team dynamics [5]. Although limitedly studied in medical and surgical context, it has been shown in a multidisciplinary gynecological setting that personality traits are associated with team performance [6]. The authors reported that negative emotionality was inversely associated with clinical teamwork scores in a communication based interaction model, essentially showing that this trait led to increased yet apparent detrimental communication. Moreover, studies in colorectal surgery have consistently shown that a surgeon’s personality influences clinical decision-making and the shared decision-making process between patient and specialist [7–9]. For example, it was identified that open-mindedness affected decision-making in a second opinion context [7]. 

Care for patients with complex vascular disease, such as aortic pathology, involves a diverse array of medical specialists, including vascular surgeons, cardiothoracic surgeons, and interventional radiologists. Oftentimes, these medical specialists work together to provide optimal care for patients, yet at the same time these specialties are regarded as independent professions with specific trades and training paradigms. The effectiveness of multidisciplinary teams does not only rely on technical expertise [9] but also importantly on the ability to communicate, collaborate, and make collective decisions. An aortic team model, founded in interdisciplinary, collaborative, and nonhierarchical partnership ensures optimal care as recently described in patients with acute and chronic conditions of the aorta [10]. A better understanding of the personality traits of stakeholders in complex multidisciplinary treatment teams could offer relevant insights into collaboration and team effectiveness, potentially giving rise to strategies for improving patient care.

The current study aims to determine the personality of medical specialists involved in the care for aortic pathologies, using the five-factor model to assess the characteristics of interventional radiologists, cardiothoracic surgeons, and vascular surgeons. This research contributes to increasing the self-awareness of surgeons about their own and others’ personality traits, which may facilitate more effective communication and collaboration. Additionally, exploring personality provides a vital foundation for decoding complex dynamics in the operating room, such as teamwork and leadership.

## Methods

### Study design and approval

The present study has been reported according to the STrengthening the Reporting of OBservational studies in Epidemiology (STROBE) guideline [11]. The study was performed in compliance with ethical and privacy regulations. The scientific committee of the department of Surgery approved the investigation prior to its distribution. The regional Medical Ethics Assessment Committee decided that the study is not subject to the Medical Research Involving Human Subjects Act (WMO).

### Respondents and personality inventory

The Dutch version of the Big Five Inventory-2 (BFI-2) was distributed among all vascular surgeons, cardiothoracic surgeons, and interventional radiologists in the Netherlands. The questionnaire was distributed online from June 2021 to January 2023 in separate instances among all general/vascular surgery departments in the Netherlands. E-mail addresses were obtained through the SUPER consortium-members, after which online anonymous links were distributed. To ensure one-time responses at the individual level, separate questionnaires were employed per hospital. Moreover, IP address-restrictions were enforced and a dedicated item for second responses included. Importantly, for interventional radiology and cardiothoracic surgery, the survey was distributed via the official communication channels of the national professional associations, respectively the Dutch Society for Thoracic Surgery (NVT) between October 2023 and March 2024 and the Dutch Society for Interventional Radiology (NVIR) between September 2023 and March 2024. In the Netherlands, all specialists are member of their respective professional association. Post-hoc quality control was performed on all individual responses, considering duration of filling in the questionnaire and response directions, and duplicates were removed. Informed consent was obtained digitally in accordance with the ethical regulations of Leiden University. Demographic data (age, gender, years of clinical experience) were collected in addition to the survey items pertaining to personality.

The validated Dutch version of the original 60-item BFI-2 includes 60 short phrases, each rated on a 1–5 Likert scale (disagree strongly – agree strongly) [12, 13]. This hierarchical model breaks down each of the five-factor trait domains into three specific facets. The choice for distribution of the 60-item BFI-2, relative to its short (-S) and extra short (-XS) versions, was based on expected response rate balanced with extensiveness of the survey. Specifically, regarding the facets in this model, open-mindedness includes aesthetic sensitivity, intellectual curiosity, and creative imagination. Conscientiousness encompasses organization, productiveness, and responsibility. Agreeableness is characterized by compassion, respectfulness, and trust. Extraversion is defined by sociability, assertiveness, and energy level. Lastly, negative emotionality includes anxiety, depression, and emotional volatility. Participants who did not complete the questionnaire were excluded from the study. Normative population data were obtained from the Longitudinal Internet studies for the Social Sciences (LISS) panel administered by Centerdata (Tilburg University, the Netherlands) [14], The LISS panel is a representative sample of Dutch individuals based on a true probability sample of households drawn from the national population register. Individuals were restricted to an age range of 35–67 to match the specialist workforce groups in the current study. The BFI-2 subset of the LISS panel was employed, as originally requested by Denissen et al. (https://osf.io/nwtx7/).

### Statistics

Analyses of covariance (ANCOVA) were conducted with Bonferroni adjustments for each set of comparisons, and gender was included as a covariate. Analyses included comparisons between 4 statistically separate groups of interest. Statistical analyses and data visualization were performed using R version 4.1.2 (R Foundation for Statistical Computing, Vienna, Austria), GraphPad Prism 8 (GraphPad Software Inc, La Jolla, CA), and Statistical Package for the Social Sciences (SPSS, version 25; IBM SPSS Inc., Armonk, NY). Qualtrics XM software (Provo, UT) was used as a survey management platform.

## Results

### Study population

A total of 224 physicians responded to the survey, including 105 vascular surgeons, 41 cardiothoracic surgeons, and 78 interventional radiologists, corresponding to response rates of 49.3% (105/213), 30.6% (41/134), and 26% (78/300), respectively (Table 2). Women represented 17.1% of vascular surgeons, 26.8% of cardiothoracic surgeons, and 20.5% of interventional radiologists. On average, vascular surgeons had 13.7 years of experience, cardiothoracic surgeons had 14.6 years, and interventional radiologists had 13.0 years of experience in clinical practice. The age distribution was similar across the groups, with vascular surgeons having a mean age of 49.9 years, cardiothoracic surgeons 49.2 years, and interventional radiologists 48.6 years. In the normative population dataset, a cohort was extracted according to the age distribution in the specialty groups (*n* = 432), the mean age was 52.1 years, and 47.9% of the respondents were female.

**Table 2:** Descriptive statistics specialist cohorts and normative population

	Normative population (*n* = 432)	Vascular surgeons (*n* = 105)	Cardiothoracic surgeons (*n* = 41)	Interventional radiologists (*n* = 78)
Age(yrs), mean (SD)	52.1 (9.2)	49.9 (8.6)	49.2 (9.9)	48.6 (9.4)
Gender identification, n (% of total) Male Female	225 (52.1) 207 (47.9)	87 (82.9) 18 (17.1)	30 (73.2) 11 (26.8)	62 (79.5) 16 (20.5)
Years of clinical experience, mean (SD)	-	13.7 (8.2)	14.6 (9.4)	13.0 (8.9)

### Personality domain variances

#### Normative population and specialist groups

**Fig. 1:**
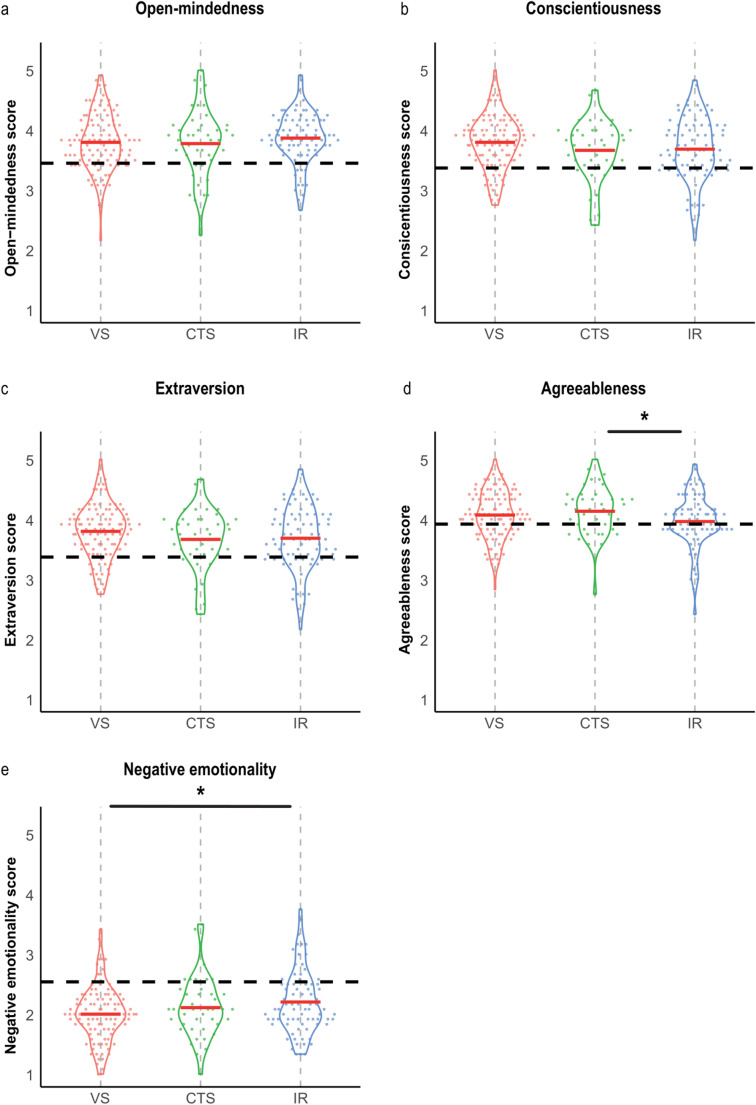
Big five personality domain comparisons between interventional and surgical specialist groups, contrasted against the normative population Personality score distributions of vascular surgeons (VS; red) cardiothoracic surgeons (CTS; green) and interventional radiologists (IR; blue) contrasted against the normative population (horizontal dashed line). (**a**) Open-mindedness, (**b**) Conscientiousness, (**c**) Extraversion, (**d**) Agreeableness, and (**e**) Negative Emotionality (scale: 1–5). Horizontal red lines represent the mean. Statistics: One-way analysis of covariance (covariate = gender, independent variable = specialty). Significance was adjusted for multiple comparisons (Bonferroni; ∗*p* <.05)

The personality domain scores of interventional and surgical specialists were contrasted with those of an age-matched general population sample (Table 3; Fig. 1).

**Table 3:** Average personality domain scores

Domain	Vascular surgeons	Cardiothoracic surgeons	Interventional radiologists
Open-mindedness, mean (SD)	3.80 (0.47)	3.78 (0.57)	3.87 (0.43)
Conscientiousness, mean (SD)	4.15 (0.45)	4.22 (0.43)	4.11 (0.49)
Extraversion, mean (SD)	3.80 (0.45)	3.66 (0.50)	3.68 (0.53)
Agreeableness, mean (SD)	4.07 (0.41)	4.13 (0.42)	4.00 (0.45)
Negative emotionality, mean (SD)	2.00 (0.45)	2.11 (0.50)	2.20 (0.53)

Across all domains, surgical and interventional specialists exhibited significant personality differences compared to the matched normative group (Fig. 1a-e). Specifically, specialists scored notably higher in open-mindedness, conscientiousness, extraversion, and agreeableness (Fig. 1a-d, *p* <.001, *p* =.002, *p* <.001, *p* <.001 *p* <.001, Supplementary Table 1). In contrast, an inverse relationship was observed for negative emotionality, with specialists scoring significantly lower than the normative population (Fig. 1e, *p* <.001, Supplementary Table 1).

#### Vascular surgeons, cardio-thoracic surgeons, and interventional radiologists

Across the five personality domains, significant differences emerged among the three specialist groups in agreeableness, and negative emotionality, while scores for open-mindedness, conscientiousness and extraversion were comparable (Fig. 1a, b, c, d and e; Table 3, Supplementary Table 2, corrected for gender). Interventional radiologists scored significantly lower on agreeableness than cardiothoracic surgeons (4.00 vs. 4.13; *p* =.046, Fig. 1d). Moreover, vascular surgeons demonstrated lower negative emotionality, scoring 2.00 compared to 2.20 for interventional radiologists (*p* =.007, Fig. 1e).

### Personality facet variances between interventional radiologists, cardiothoracic surgeons, and vascular surgeons

To provide more insight into the personality structure of the interventional and surgical specialist groups, we performed analyses on all three subordinate facets within each of the big five domains (Table 1).

**Fig. 2:**
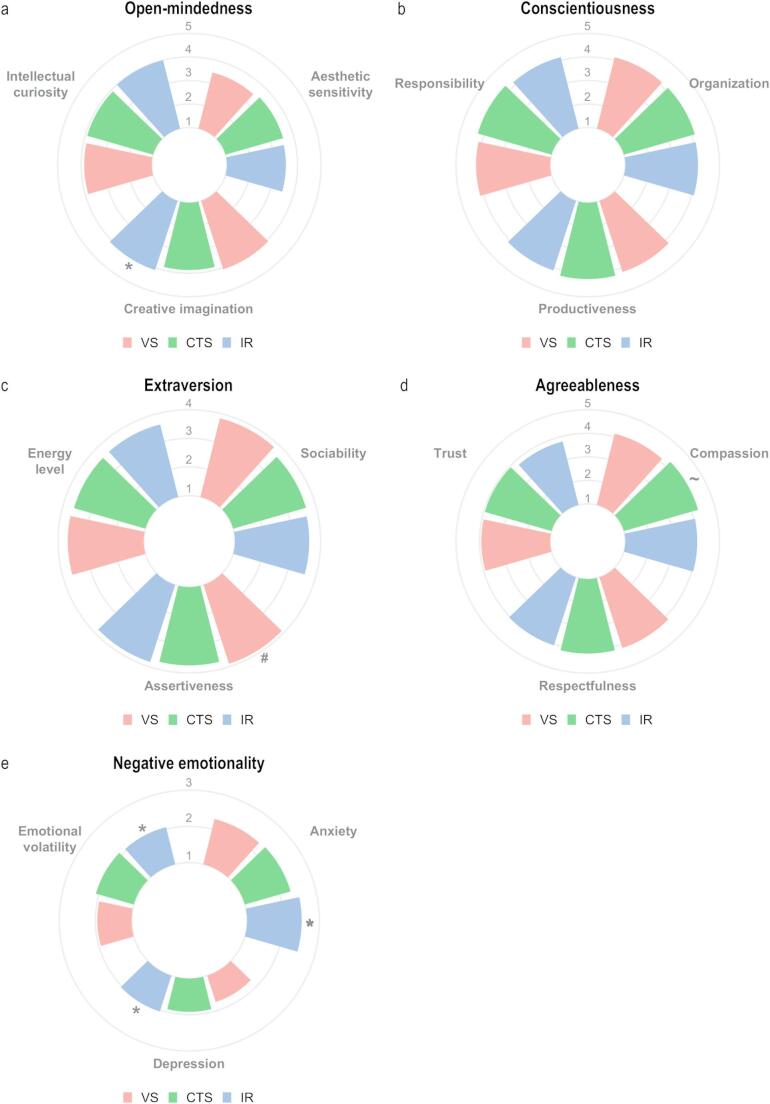
Trait facet averages of personality scores of interventional and surgical specialist groups. Within each of the five domains of personality, three subdomains (facets) may be distinguished. Here, average facet scores are visualized for vascular surgeons (red), cardiothoracic surgeons (green), and interventional radiologists (blue) per domain; (**a**) Open-mindedness, (**b**) Conscientiousness, (**c**) Extraversion, (**d**) Agreeableness, and (**e**) Negative Emotionality. Statistics: One-way analysis of covariance (covariate = gender). The mean difference is based on estimated marginal means and significance was adjusted for multiple comparisons (Bonferroni; significantly different (*p* <.05) versus interventional radiologists (IR; *), cardiothoracic surgeons (CTS; ~), and vascular surgeons (VS; #))

In open-mindedness, consisting of intellectual curiosity, aesthetic sensitivity, and creative imagination, significant differences were observed for the latter facet. More specifically, interventional radiologists appeared to score significantly higher (4.10) in creative imagination relative to cardiothoracic surgeons (3.87, *p* =.037, Fig. 2a). For the three facets of conscientiousness, no significant differences were observed (Fig. 2b). In extraversion, significant differences were observed for the facet of assertiveness, with vascular surgeons showing higher average scores relative to interventional radiologists (3.83 vs. 3.60, *p* =.005, Fig. 2cInterventional radiologists scored significantly lower on compassion (4.06), a facet of agreeableness, relative to cardiothoracic surgeons (4.28, *p* =.022), Fig. 2d). Within negative emotionality, significant differences were observed in anxiety, depression, but not in emotional volatility. Notably, vascular surgeons scored significantly lower on depression (1.77 vs. 2.04, *p* <.001) and anxiety (2.29 vs. 2.51, *p* =.013) as compared to interventional radiologists, whereas no significant differences were observed relative to cardiothoracic surgeons (Fig. 2e).

## Discussion

We comprehensively studied the personality of three medical specialties involved in shared clinical pathways for treatment of complex vascular disease such as aortic pathology. Interventional radiologists, vascular surgeons, and cardiothoracic surgeons demonstrated a distinct personality profile relative to the general population, while differences were also present between the specialty groups. Specifically, domain variations were found in agreeableness and negative emotionality. A deeper analysis of subdomains uncovered finer distinctions in personality facets like assertiveness, compassion, creative imagination, and emotional volatility.

Previous personality research in medical specialties has often focused on broad categorizations, such as medical versus surgical disciplines [2, 3, 15]. Our study provides new insights into detailed personality traits of specialized groups involved in a shared clinical pathway, while also showing differences in personality relative to the normative population. Although surgical and interventional specialty groups have not yet been thoroughly investigated for personality traits, the relative distinction of our cohort’s personality profile to the general population aligns with previous findings [1, 16, 17]. Specifically, interventional and surgical specialists tend to score higher on open-mindedness, conscientiousness, extraversion, and lower on negative emotionality relative to the general population. To the best of our knowledge, no comprehensive studies have previously been published on the personality of vascular surgeons and interventional radiologists. However, a British study among 261 consultant cardiac surgeons was consistent with our results, showing higher conscientiousness and agreeableness and lower negative emotionality in surgeons compared to the general population [18]. The observed differences between the various specialty groups in this study may result from self-selection effects during the student and early physician phases, where individual personality traits and skill sets not only align naturally with certain specialties but also generate enthusiasm among mentors and within the broader specialty community [19]. Specialty-specific dynamics likely also contribute to the reinforcement of average personality profiles over time, although additional factors such as generational preferences, demographic changes, and professional developments should also be considered [20]. Importantly, the observed variability within the specialist groups might be of interest in collaborative settings, self-development, and interpersonal interactions.

Caring for patients with complex cardiovascular disease requires multidisciplinary expertise and collaboration across various medical specialties. Unlike unidisciplinary teams, where members can attract and select new recruits, multidisciplinary teams consist of specialists from different fields who collaborate without directly regulating team composition. In practice, this collaboration involves shared clinical ward duties, multidisciplinary team meetings, coordination of clinical decisions, and joint surgeries or interventions. Moreover, medical specialists in multidisciplinary teams should be flexible, empathetic, and aware of themselves and others to facilitate shared decision-making between the team and their patients. The current study illustrates that specialist teams caring for complex pathology (such as aortic pathology) may consist of individuals with diverse personality attributes. These personality differences exist both across and within specialties, underscoring the complexity of this multidisciplinary teamwork and recognizing the existence of varied team compositions.

A prerequisite for effective teamwork is psychological safety, which is defined as an environment in which there is a shared belief among team members that it is safe to take interpersonal risks (i.e., in which team members are comfortable to share any thoughts, ideas or concerns) [22–24]. There is a conceivable relationship between psychological safety at work and personality factors. Capabilities such as leadership role modelling, active listening, and facilitating inclusivity, in addition to open-mindedness and interpersonal awareness are essential to creating a safe environment. It is well known that psychologically safe environments are associated with enhanced team performance [22, 25]. Teams that feel psychologically safe are more likely to speak up about potential errors, share innovative ideas for problem-solving, and adapt effectively to changing situations during procedures. As a result, psychological safety does not only optimize teamwork but also minimizes preventable errors, improves adherence to safety protocols, and ultimately enhances patient care and clinical outcomes.

When focusing on team performance, Shared Mental Models (SMM) reflect the collective understanding of teams about their functioning and dynamics, including interpersonal interactions, awareness, and responsibilities. In short, SMM, as a cognitive construct among medical teams, characterize how team members engage with their professional environment and undertake difficult challenges [26–28]. Taking into account such shared constructs, acknowledging that personality traits have distinct implications depending on the situational context is essential; for example, negative emotionality may increase danger awareness, and disagreeableness may facilitate quicker decision-making [29]. Likewise, higher levels of agreeableness throughout the team may facilitate common understanding of members’ strengths, weaknesses, preferences, and behaviors. Imaginably, a mutual understanding of these personality traits can enhance the development of a robust SMM, improving operational efficiency, interpersonal communication, optimizing task distribution, and effectively utilizing members’ talents, all of which contribute to more effective team performance. When translating these findings to the clinical setting, it might be of interest to combine personality with teamwork simulation frameworks and encompassing scales [6]. 

Ideally, insights into personality traits on a specialty-wide, or personal level, can aid in becoming or growing as a high-performance team. This extends beyond medical specialists to include a broader spectrum of health professionals such as nurses, caregivers, and paramedics. The mix of educational backgrounds and professional responsibilities in these interdisciplinary teams not only brings diverse viewpoints but also results in palpable differences in role-based responsibilities. These dynamics can sometimes lead to friction, yet they also offer significant opportunities for enhancing collaborative practices and improving team effectiveness. For instance, methodologies such as Team Based Learning, which focus on small group learning through teamwork and direct feedback, might be able to incorporate personality traits, further enhancing collaboration and overall team performance [30, 31]. Another approach could be to organize intervision sessions among teams, incorporating personal characteristics specifically [32]. The concept of intervision aims to achieve peer reflection by discussing experiences among colleagues, guided by a coach, which has been shown to be valuable for, among others, identity development and solution-oriented working. Lastly, the established approach of joint problem-solving orientation, as highlighted by Kerrissey et al., may be of value [33]. In this context, viewing problems as shared and solutions to require co-production were suggested to improve the effectiveness of cross-boundary teams. One can imagine that mutual understanding on a personal level, considering each other’s personalities, builds rapport and creates more dynamic and cohesive teams. Ultimately, such integrations may result in improved patient outcomes and increased long-term job satisfaction [34]. 

All in all, this study shows the importance of creating awareness about the role of personality in surgical teams. As personality differences can affect various factors on a team-level (such as team learning, psychological safety, and joint-problem solving orientation), team members have the responsibility to propagate flexibility toward possible (personality) differences they might have, in order to facilitate the collaboration and optimal training of residents. Integration of such insights toward a clinical setting might possibly be facilitated via postgraduate education programs or integrated with existing methodologies, yet before translation the next step should be to investigate how personality characteristics relate to other established determinants of team effectiveness, such as psychological safety.

### Strengths and limitations

The design of our study encompasses several strengths and limitations. Intentional response distortion and self-reporting bias on the questionnaire, although recognized, is unlikely given the absence of external pressures. The inclusion of all surgical departments in the Netherlands, together with dissemination through the national societies, allows for statistical power and mitigates potential selection bias. The authors are additionally aware and of potential non-response selection bias in the current study, inherent to a response rate < 100%. Considering the slightly elevated proportion of female respondents in the interventional radiology and cardiothoracic surgery groups, we have accounted for gender differences by applying statistical corrections to mitigate biases related to sex in our analysis. Lastly, while this study primarily identifies differences in personality dimensions among specialty groups, further research is needed to explore how these traits directly influence collaborative interactions.

## Conclusion

In summary, our study explores the Big Five personality traits of three distinct specialties involved in a multidisciplinary care pathway. We found distinct personality profiles of interventional radiologists, cardiothoracic surgeons, and vascular surgeons compared to the general population and noted differences between these specialties, particularly in agreeableness and negative emotionality. This research enhances our understanding of personality within the context of shared clinical care and sparks potential research initiatives geared toward building effective multidisciplinary teams.

## Supplementary Information

Below is the link to the electronic supplementary material.

Supplementary Material 1 (DOCX 25.4 KB) 

## Data Availability

The data that support the findings of this study are available in the supplementary material. The individual-level data are not publicly available considering privacy or ethical restrictions and are stored and safeguarded accordingly.
