# Supplementary material for: Cut from the same cloth? investigating the personality of interventional and surgical cardiovascular specialists
Source: Langenbecks Arch Surg. 2025 Oct 23;410(1):309. doi: 10.1007/s00423-025-03874-7 (PMC12549727; doi:10.1007/s00423-025-03874-7)
Supplement: Supplementary file 1 — Supplementary Material 1 (DOCX 25.4 KB) [file 423_2025_3874_MOESM1_ESM.docx]

**Supplementary Table 1**

| **Pairwise Comparisons** | | | | | | | |
| --- | --- | --- | --- | --- | --- | --- | --- |
| Dependent Variable | (I) Group | (J) Group | Mean Difference (I-J) | Std. Error | Sig.^b^ | 95% Confidence Interval for Difference^b^ | |
|  |  |  |  |  |  | Lower Bound | Upper Bound |
| Extraversion | Normative population | Vascular surgeon | -,423^*^ | ,063 | <,001 | -,589 | -,256 |
|  |  | Cardiothoracic surgeon | -,359^*^ | ,076 | <,001 | -,560 | -,159 |
|  |  | Interventional radiologist | -,306^*^ | ,067 | <,001 | -,484 | -,128 |
| Agreeableness | Normative population | Vascular surgeon | -,181^*^ | ,050 | ,002 | -,313 | -,049 |
|  |  | Cardiothoracic surgeon | -,229^*^ | ,060 | <,001 | -,387 | -,070 |
|  |  | Interventional radiologist | -,068 | ,053 | 1,000 | -,209 | ,073 |
| Conscientiousness | Normative population | Vascular surgeon | -,402^*^ | ,057 | <,001 | -,552 | -,252 |
|  |  | Cardiothoracic surgeon | -,473^*^ | ,068 | <,001 | -,653 | -,292 |
|  |  | Interventional radiologist | -,372^*^ | ,061 | <,001 | -,532 | -,211 |
| Negative emotionality | Normative population | Vascular surgeon | ,478^*^ | ,063 | <,001 | ,312 | ,643 |
|  |  | Cardiothoracic surgeon | ,356^*^ | ,075 | <,001 | ,157 | ,555 |
|  |  | Interventional radiologist | ,301^*^ | ,067 | <,001 | ,123 | ,478 |
| Open-mindedness | Normative population | Vascular surgeon | -,336^*^ | ,063 | <,001 | -,502 | -,171 |
|  |  | Cardiothoracic surgeon | -,314^*^ | ,075 | <,001 | -,514 | -,115 |
|  |  | Interventional radiologist | -,405^*^ | ,067 | <,001 | -,582 | -,228 |
| Based on estimated marginal means | | | | | | | |
| *. The mean difference is significant at the ,05 level. | | | | | | | |
| b. Adjustment for multiple comparisons: Bonferroni. | | | | | | | |

**Supplementary Table 2**

| **Pairwise Comparisons** | | | | | | | |
| --- | --- | --- | --- | --- | --- | --- | --- |
| Dependent Variable | (I) Medical specialist | (J) Medical specialist | Mean Difference (I-J) | Std. Error | Sig.^b^ | 95% Confidence Interval for Difference^b^ | |
|  |  |  |  |  |  | Lower Bound | Upper Bound |
| Extraversion | Vascular surgeon | Cardiothoracic surgeon | ,162 | ,099 | ,104 | -,034 | ,357 |
|  |  | Interventional radiologist | ,127 | ,076 | ,096 | -,023 | ,276 |
|  | Cardiothoracic surgeon | Vascular surgeon | -,162 | ,099 | ,104 | -,357 | ,034 |
|  |  | Interventional radiologist | -,035 | ,097 | ,716 | -,226 | ,156 |
|  | Interventional radiologist | Vascular surgeon | -,127 | ,076 | ,096 | -,276 | ,023 |
|  |  | Cardiothoracic surgeon | ,035 | ,097 | ,716 | -,156 | ,226 |
| Agreeableness | Vascular surgeon | Cardiothoracic surgeon | -,102 | ,085 | ,234 | -,270 | ,066 |
|  |  | Interventional radiologist | ,088 | ,065 | ,176 | -,040 | ,217 |
|  | Cardiothoracic surgeon | Vascular surgeon | ,102 | ,085 | ,234 | -,066 | ,270 |
|  |  | Interventional radiologist | ,190^*^ | ,083 | ,024 | ,026 | ,355 |
|  | Interventional radiologist | Vascular surgeon | -,088 | ,065 | ,176 | -,217 | ,040 |
|  |  | Cardiothoracic surgeon | -,190^*^ | ,083 | ,024 | -,355 | -,026 |
| Conscientiousness | Vascular surgeon | Cardiothoracic surgeon | -,105 | ,093 | ,258 | -,288 | ,078 |
|  |  | Interventional radiologist | ,020 | ,071 | ,780 | -,120 | ,159 |
|  | Cardiothoracic surgeon | Vascular surgeon | ,105 | ,093 | ,258 | -,078 | ,288 |
|  |  | Interventional radiologist | ,125 | ,091 | ,169 | -,054 | ,304 |
|  | Interventional radiologist | Vascular surgeon | -,020 | ,071 | ,780 | -,159 | ,120 |
|  |  | Cardiothoracic surgeon | -,125 | ,091 | ,169 | -,304 | ,054 |
| Negative emotionality | Vascular surgeon | Cardiothoracic surgeon | -,058 | ,098 | ,552 | -,251 | ,134 |
|  |  | Interventional radiologist | -,182^*^ | ,075 | ,015 | -,329 | -,035 |
|  | Cardiothoracic surgeon | Vascular surgeon | ,058 | ,098 | ,552 | -,134 | ,251 |
|  |  | Interventional radiologist | -,124 | ,096 | ,197 | -,312 | ,065 |
|  | Interventional radiologist | Vascular surgeon | ,182^*^ | ,075 | ,015 | ,035 | ,329 |
|  |  | Cardiothoracic surgeon | ,124 | ,096 | ,197 | -,065 | ,312 |
| Open-mindedness | Vascular surgeon | Cardiothoracic surgeon | ,009 | ,097 | ,930 | -,184 | ,201 |
|  |  | Interventional radiologist | -,077 | ,074 | ,303 | -,223 | ,070 |
|  | Cardiothoracic surgeon | Vascular surgeon | -,009 | ,097 | ,930 | -,201 | ,184 |
|  |  | Interventional radiologist | -,085 | ,095 | ,371 | -,273 | ,102 |
|  | Interventional radiologist | Vascular surgeon | ,077 | ,074 | ,303 | -,070 | ,223 |
|  |  | Cardiothoracic surgeon | ,085 | ,095 | ,371 | -,102 | ,273 |
| Based on estimated marginal means | | | | | | | |
| *. The mean difference is significant at the ,05 level. | | | | | | | |
| b. Adjustment for multiple comparisons: Least Significant Difference (equivalent to no adjustments). | | | | | | | |
